# Evidence and sources of placebo effects in transcranial direct current stimulation during a single session of visuospatial working memory practice

**DOI:** 10.1038/s41598-024-59927-2

**Published:** 2024-04-20

**Authors:** Andrew Hooyman, Nicole K. Haikalis, Peiyuan Wang, Heidi M. Schambra, Keith R. Lohse, Sydney Y. Schaefer

**Affiliations:** 1 School of Biological and Health Systems Engineering, Arizona State University, 501 E. Tyler Mall, MC 9709, Tempe, AZ 85287 USA; 2 Department of Neurology and Department of Physical Medicine and Rehabilitation, Grossman School of Medicine, New York University, New York, NY USA; 3 Program in Physical Therapy, Washington University School of Medicine, St. Louis, MO USA

**Keywords:** Health occupations, Medical research

## Abstract

Transcranial direct current stimulation (tDCS) can be used to non-invasively augment cognitive training. However, the benefits of tDCS may be due in part to placebo effects, which have not been well-characterized. The purpose of this study was to determine whether tDCS can have a measurable placebo effect on cognitive training and to identify potential sources of this effect. Eighty-three right-handed adults were randomly assigned to one of three groups: control (no exposure to tDCS), sham tDCS, or active tDCS. The sham and active tDCS groups were double-blinded. Each group performed 20 min of an adapted Corsi Block Tapping Task (CBTT), a visuospatial working memory task. Anodal or sham tDCS was applied during CBTT training in a right parietal-left supraorbital montage. After training, active and sham tDCS groups were surveyed on expectations about tDCS efficacy. Linear mixed effects models showed that the tDCS groups (active and sham combined) improved more on the CBTT with training than the control group, suggesting a placebo effect of tDCS. Participants’ tDCS expectations were significantly related to the placebo effect, as was the belief of receiving active stimulation. This placebo effect shows that the benefits of tDCS on cognitive training can occur even in absence of active stimulation. Future tDCS studies should consider how treatment expectations may be a source of the placebo effect in tDCS research, and identify ways to potentially leverage them to maximize treatment benefit.

## Introduction

Visuospatial working memory is the cognitive capacity to maintain a representation of visuospatial information for a brief period. Visuospatial working memory deficits can significantly impair daily life^1^ and can emerge in a number of neurological conditions^2^. Transcranial direct current stimulation (tDCS) has been used to augment or improve cognitive training in the visuospatial working memory domain^3^. The mechanism by which tDCS is thought to modify behavior is by changing cortical electrical fields^4^, which at the cellular level impact membrane potentials leading increased spike timing precision^5^. For example, anodal tDCS applied over the prefrontal cortex in humans during visuospatial training results in within-session gains in performance^6,7^, with similar results in rat models^8^. Visuospatial function has also been enhanced by tDCS in clinical populations, such as those with depression^9^ or history of stroke^10^. However, the efficacy and reliability of tDCS to modulate either the targeted neural circuitry or behavior is mixed. As a result, the field of non-invasive brain stimulation has generated several consensus statements that provide recommendations on how to utilize brain stimulation to improve consistency and reliability between studies^11–13^. However, missing among these recommendations are ways to measure and control for placebo-related factors.

A placebo effect is the beneficial effect on outcomes due to the perception of a treatment, rather than from the actual treatment itself^14^. One possible factor contributing to placebo effects of tDCS is participants’ expectations about the efficacy of stimulation^15^. Expectations about the efficacy of tDCS as a treatment tend to be positive on average, but range from very skeptical to very positive^16^. Thus, in a sham-controlled tDCS study, it is possible that higher expectations could be unknowingly over-sampled in either the sham group or the active tDCS group. Consequently, null results in any given tDCS study could, in part, be due to higher expectations (and therefore larger placebo effects) in the sham group that end up masking treatment effects in the active tDCS group. Conversely, positive effects of active tDCS could also (unknowingly) due to significantly higher expectations in the active group compared to the sham group. These scenarios are plausible especially for small sample sizes, which are common in tDCS research^17^. To demonstrate the potential strength of a placebo effect due to participant expectations of tDCS on cognitive training, Rabipour et al. verbally primed participants with either positive or negative information about tDCS to increase or decrease their expectations. Following a single 20-min session of a custom n-back task, executive function performance was greatest in people who had been primed to have high expectations of tDCS, irrespective of stimulation condition, suggesting a possible benefit of high expectations over stimulation. They also found that participants who were primed to have low expectations of tDCS showed no improvement on the cognitive tests, irrespective of active or sham stimulation. As such, Rabipour et al. argued that clinical trials “may succeed or fail due to people’s pre-existing beliefs regarding the effectiveness of treatment received”.

The purpose of this study was to examine how participants’ expectations of tDCS impacted how much they improved on a visuospatial memory task (namely, the Corsi Block Tapping Task) over the course of a single session of training concurrent with double-blind tDCS. Based on theories of placebo effects and previous studies^15,18,19^, we hypothesized that higher expectations of tDCS would be associated with more improvement, regardless of whether they received active or sham tDCS.

## Results

As shown in Table 1, there were no significant differences between the control, sham tDCS, and active tDCS groups in terms of age (F(2,80) = 0.14, p = 0.87), sex (χ^2^(2) = 4.80, p = 0.09), number of trials completed during training (F(2,80) = 1.33, p = 0.27), trial duration (F(2,80) = 0.37, p = 0.69), or trial accuracy (F(2,80) = 0.15, p = 0.86). Furthermore, there was no significant difference in the expectations of tDCS reported by the active and sham tDCS groups (t(54) = 1.40, p = 0.20), as well as no significant difference in the proportion of participants who perceived receiving active tDCS, sham tDCS, or were not sure (χ^2^(2) = 0.46, p = 0.80). During data collection two participants in the Sham group and one participant in the Active group did not complete the tDCS questionnaire that is described below in “Methods”.

**Table 1:** Participant characteristics and training for the control, active tDCS, and sham tDCS groups.

Measure	Control group	Active tDCS group	Sham tDCS group	p-value
Age	22.48 (4.30)	22.03 (4.51)	22.58 (3.97)	0.87
Sex (F/M)	15/8	11/18	12/19	0.09
# Training trials completed	64.08 (10.09)	58.34 (15.03)	60.25 (13.21)	0.27
Average trial duration—seconds	18.63 (1.99)	18.24 (1.36)	18.52 (1.81)	0.69
Trial accuracy	66.74 (0.07)	65.98 (0.11)	65.36 (0.09)	0.86
Expectation^a^	–	5.41 (1.93)	4.69 (1.98)	0.20
Perceived stimulation condition^b^				
Active	–	16	14	0.80
Sham	–	3	4	
Not sure	–	9	11	

All values are reported as mean (SD) unless otherwise noted.

^a^Measured on a Likert scale of 0–8, where higher values indicate higher expectations of tDCS.

^b^Counts of participants per group per perceived stimulation condition.

### No significant treatment effect of active tDCS

**Figure 1:**
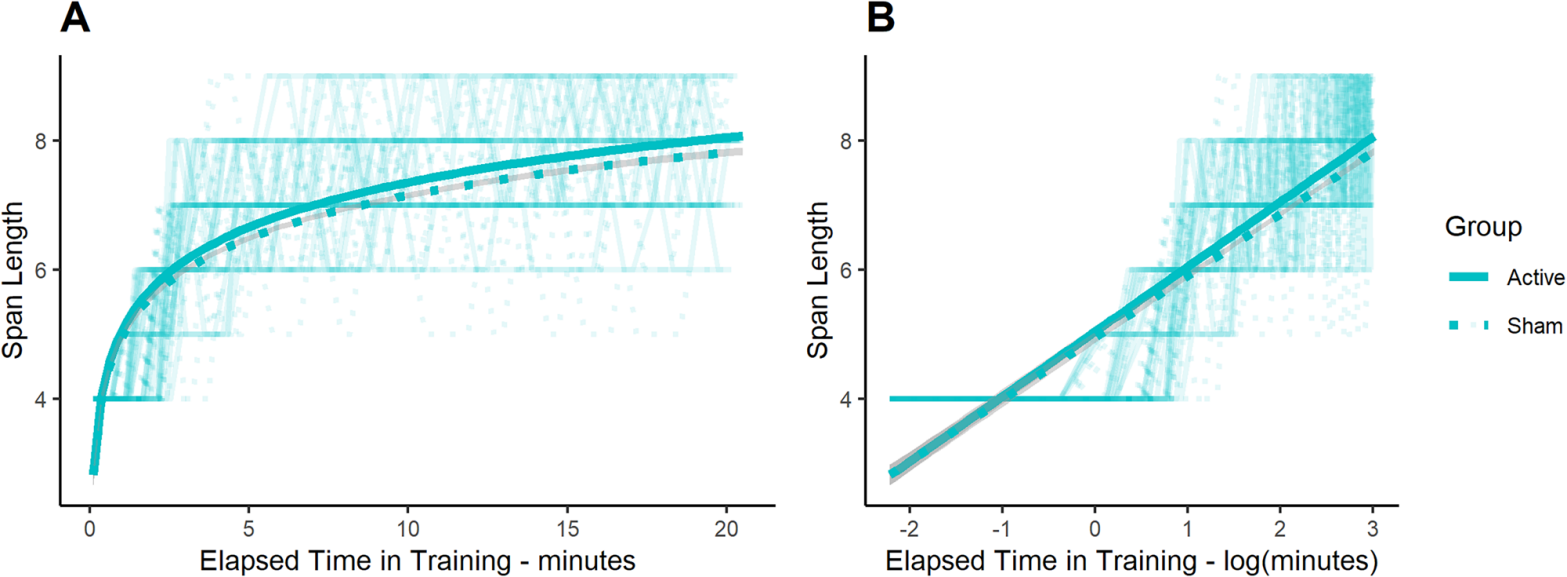
(**A**) Span length as a function of time in training for the active tDCS group (solid line) and the sham tDCS group (dotted line), fitted to the individual data. Gray regions indicate standard error. (**B**) Span length as a function of log time in training for the active tDCS group (solid line) and the sham tDCS group (dotted line), fitted to the individual data. Note how when the model is fit to the log-transformed time series (as shown in panel **B**), the resulting fits are linear. Gray regions indicate standard error.

Our linear mixed effects model showed no main effect of group (β_sham_ = − 0.07, t(58) =  − 0.24, p = 0.81) on CBTT performance, which was measured as span length (active tDCS mean ± SD: 6.95 ± 1.38, sham tDCS: 6.74 ± 1.39). There was also no significant log time in training-by-group interaction on span length (β = − 0.005, t(58) =  − 0.23, p = 0.82) (Fig. 1). Given this non-significant difference between groups, we performed a follow-up equivalence test^20^. To determine the upper and lower bounds of our equivalence test, we chose an interval that equated to a mean difference between groups of 0.50. With the pooled standard deviation between the groups of 1.41, we determined that the lower bound of our equivalence test was -0.75 and the upper bound was 0.75. Thus, any mean difference between groups that was a span length ± 0.75 would be considered a moderate and meaningful effect size. The results of the equivalence test between the active and sham groups were not significant (t(58) =  − 1.51, p = 0.07). Generally speaking, a non-significant equivalence test implies that a difference between groups is not meaningful or worthwhile^20^.

### Evidence of significant placebo effect of tDCS

**Figure 2:**
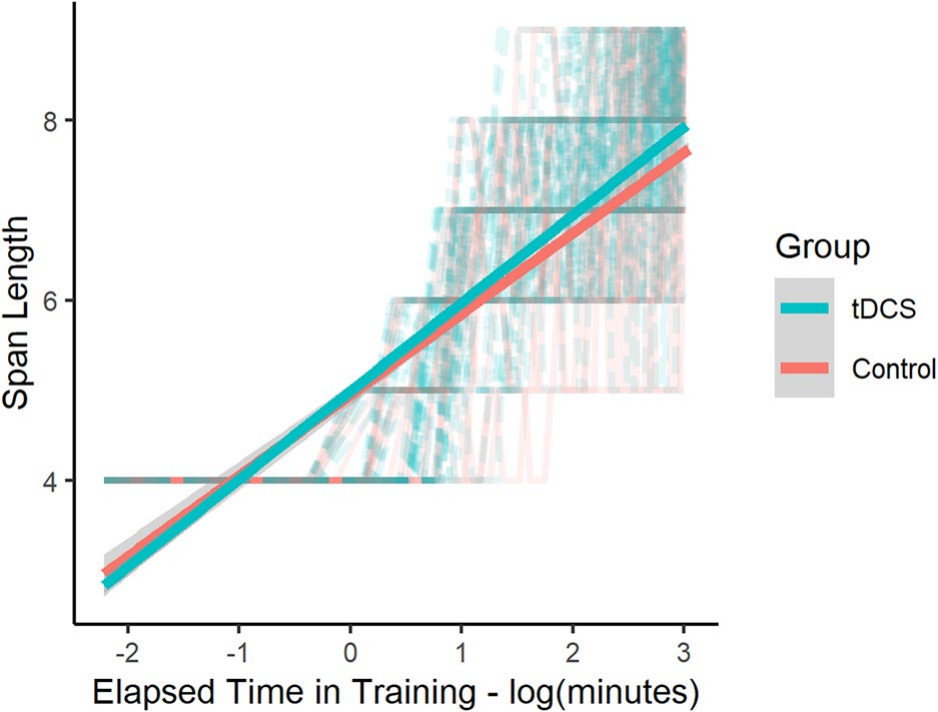
Span length as a function of log time in training for the combined tDCS groups (active and sham, blue line) and the no-tDCS control group (red line), fitted to the individual data. Gray regions indicate standard error.

Our linear mixed effects model examining the presence of a placebo effect showed that both the sham (β = 0.46, t(82), p = 0.02) and active (β = 0.50, t(82), p = 0.04) tDCS groups performed better than the control group (mean ± SD: 6.65 ± 1.48). When collapsed across tDCS groups and then compared to the control group, there was a significant log time in training-by-group interaction (β = 0.06, t(5125) = 3.42, p < 0.01), suggesting that the participants who were exposed to the tDCS device improved more on the CBTT than the control group (who had no exposure to the device) (Fig. 2). Interestingly, there was also a main effect of group (control vs. tDCS) where individuals who were exposed to the tDCS device (either during active or sham stimulation) had a slightly lower initial best span compared to the controls (β = − 0.73, t(83) =  − 2.49, p = 0.01). Altogether, this result indicates that although control participants had better initial performance on the task, with continued practice, participants who were exposed to tDCS demonstrated superior performance to that of the controls. There was no effect of sex on CBTT performance across these three groups (β = 0.25, t(83) = 1.78, p = 0.08).

### Effects of tDCS expectation and perceived stimulation on CBTT training

**Figure 3:**
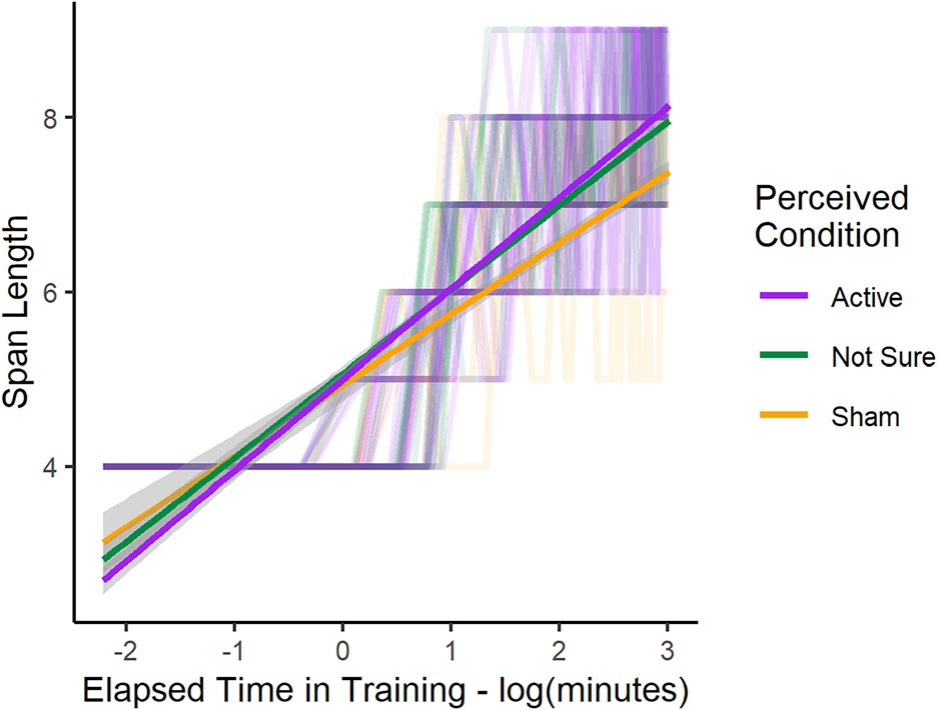
Span length as a function of log time in training based on perceived group assignment (Active: purple, Not Sure: green, and Sham: yellow), fitted to the individual data. Gray regions indicate standard error.

Our analyses revealed a main effect of expectation on span length, such that higher expectations of tDCS to improve task performance were associated with longer span lengths on average (β = 0.05; t(53) = 2.20 p = 0.04); thus, a 2.8 point difference in expectation between participants could account for the mean difference in span length between the tDCS and control groups. For reference, expectations were self-reported on a 0 to 8 scale (see “Methods”). There was also a main effect of perceived stimulation condition, where individuals who were either not sure or believed they received active tDCS (not sure: 6.83 ± 1.36, p = 0.03; active: 6.91 ± 1.39; p = 0.03) had higher span lengths than those who believed they received sham tDCS (6.34 ± 1.46) (Fig. 3). There was also a significant log time in training-by-belief interaction (F(2,3305) = 16.80, p < 0.001), where individuals who were not sure and those who believed they received active tDCS improved performance at a faster rate than those who believed they received sham tDCS (not sure: β = 0.15, t(3305) = 3.99, p < 0.001, active: β = 0.20, t(3305) = 5.73, p < 0.001).

**Figure 4:**
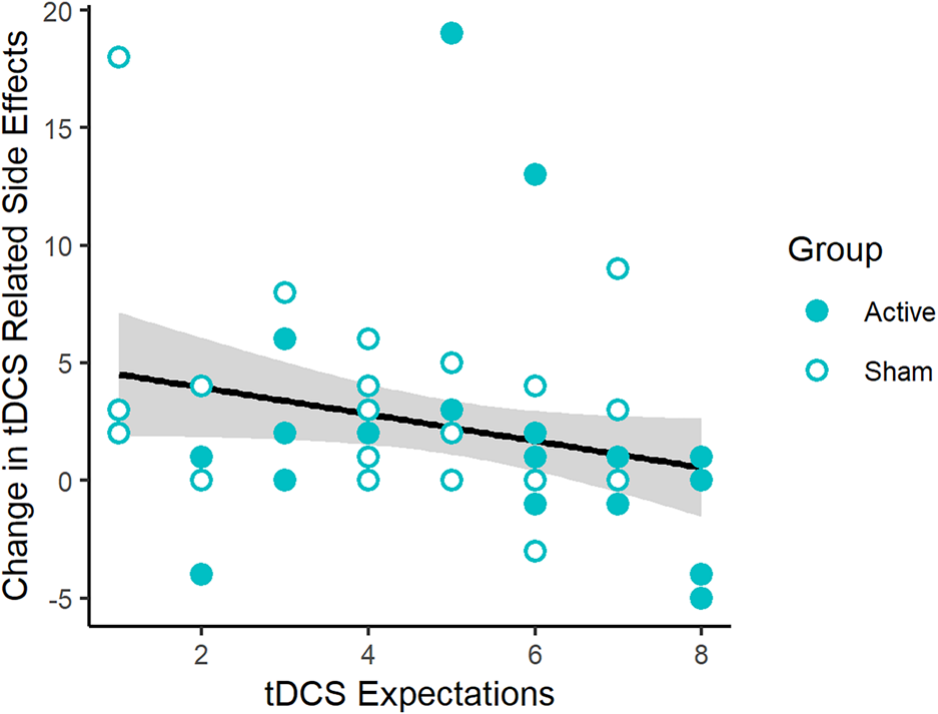
Changes in total symptom score on the tDCS Questionnaire from pre- to post-stimulation for the active tDCS group (closed circle) and sham tDCS group (open circle), as a function of tDCS expectations, where higher values indicate more belief that tDCS can improve cognitive function. Gray regions indicate standard error.

We also used a linear model to determine whether changes in negative symptoms from pre- to post-stimulation were predicted by group, expectation that tDCS will improve cognitive function, and perceived stimulation condition. Results demonstrated that neither group (β_sham_ = 0.73, t(51) = 0.64, p = 0.52) nor perceived stimulation condition (F(2,51) = 2.6, p = 0.08) had a significant effect on whether negative symptoms (e.g., headache, neck pain) changed from pre- to post-stimulation. However, there was a significant main effect of expectation on how much symptoms changed from before to after the tDCS session (β = − 0.63; t(51) =  − 2.16, p = 0.04) (Fig. 4).

## Discussion

The purpose of this study was to examine how participants’ expectations of tDCS improved a visuospatial memory task (the CBTT) over the course of a single session of training. We found no effect of active (anodal) tDCS on visuospatial performance (span length), in keeping with other studies reporting null findings^21–23^. Given our study design, we were able to show that relative to a control group that did not have any exposure to the tDCS device, groups that did have exposure to the device (either through sham or active tDCS) have better CBTT performance overall, as well as more improvement on the CBTT with practice. These results support the presence of a placebo effect where both sham and active groups perform better than the control group, which was related to participants’ expectations of tDCS to improve cognitive performance. We found that participants with high expectations of tDCS tended to improve more on the CBTT than participants with lower expectations, and tended to experienced fewer negative symptoms following stimulation. We also confirmed that blinding was effective in this study, given that both tDCS groups could not reliably report which condition they actually received.

While placebo effects of tDCS have been shown to improve pain (e.g.,^24–26^), to our knowledge this is one of the first studies besides Rabipour et al. to not only demonstrate a placebo effect of tDCS on cognitive training, but to also show that the magnitude of that placebo effect on cognitive training is related to expectation amount. Previous research using tDCS to enhance processing speed (measured via serial reaction time) has shown that inert electrical stimulation can improve performance more than a control group, but has not identified sources of this effect, such as expectations^27^. Rabipour et al. showed that priming expectations through positive (or negative) information about tDCS prior to stimulation can improve (or not improve) executive function post-stimulation^15^. Although here we took steps to ensure that we did not prime participants in advance of the study (e.g., using a script, asking the expectation question after stimulation), the association of lower tDCS expectations with lower visuospatial working memory performance during training are nevertheless consistent with Rabipour et al.’s study. This previous study by Rabipour et al. also showed more negative symptoms (higher levels of itchiness and heat at electrode site) associated with lower expectations, consistent with our results in Fig. 4. Recent research has shown that the severity of negative side effects related to tDCS can be manipulated based on framing of the likelihood of a negative effect occurring^28^. This suggests that the presence and/or magnitude of negative side effects of tDCS may be mitigated through priming or inducing a positive mood^29^.

The current study also showed that the belief of receiving sham tDCS was associated with worse performance on the CBTT, thereby driving what has been referred to in the literature as a ‘lessebo’ effect^30–34^. This phenomenon has been described when participants have an attenuated or diminished effect from an intervention because they believe that they did not receive the intervention, relative to participants who believe they received the intervention. This result highlights how important participant blinding is to minimize a placebo effect. Optimal blinding can be facilitated through, for example, using a script to ensure the same information is given to all participants so as to minimize any influence on participant expectations about tDCS or the treatment they may receive. Furthermore, other blinding techniques in regard to sham stimulation, such as double-peak stimulation, concurrent use of local anesthesia, or stimulation of irrelevant area to control for sensory effects, could also be utilized to reduce participant perception of group assignment^35^. We note, however, that we did not record how long it took to set up each participant with the tDCS device. It is plausible that some participants had lengthier set-up times than others, adding inter-subject variability in the testing environment in terms of hands-on time with the experimenters. All experimenters followed the same script though, and were instructed to not engage with the participants beyond the established protocol.

Previous research aimed at augmenting cognitive training with tDCS has struggled with mixed findings that have not fully supported the use of tDCS to reliably affect behavior or modulate cortical excitability^21^, which may in part be due to uncontrolled placebo effects. Thus, there is room to improve best practices within tDCS research to control for the potential sources reported here, namely participant expectations and beliefs about whether stimulation was received. Although a sham-controlled, double-blinded trial is currently the gold standard, if the two groups (active and sham) are very small (n < 12) (which is common in human tDCS research, according to the open-access tDCS database^17^), then the likelihood of having a significant difference in expectations between the two groups is higher than with larger sample sizes closer to n = 30. Should this unknowingly occur and expectations are not collected in a given study, then one cannot rule out that a significant difference in the primary variable between the active and sham tDCS groups.

As such, future work should include ways to quantify and control for potential placebo effects to better understand the mechanisms of tDCS as an intervention during cognitive training and beyond. Data from this study support measuring participant expectations and perceived stimulation conditions to control for potential sources of placebo effects. However, if the goal of tDCS as an intervention is to improve overall patient outcomes, then future research should also consider ways to harness, leverage, or exploit placebo effects to boost any potential treatment effects that would theoretically be layered on top of (but are functionally distinct from) any stimulation effect.

## Methods

### Participant characteristics and group assignment

The sample size for this study was computed with G-Power 3.1^36^. Based on our initial pilot data and previous literature investigating non-invasive brain stimulation placebo effects on motor and cognitive outcomes^27,37^, we estimated an effect size of f = 0.25. Given a repeated measures design with interactions with 3 groups, α = 0.05, power of 0.95, correlation among repeated measures of 0.2, and non-sphericity correction of 1, we estimated a total sample size of 84. All participants in this study had no self-reported history of mental illness, neurologic disease, or injury (i.e., stroke, history of seizures, concussion diagnosis, brain disease, or arthritis of the hands or upper limbs). All participants reported normal visual acuity and the absence of any peripheral sensory or motor loss/pathology. Eighty-three right-handed participants were recruited into this study (mean age = 22.2; 38 females). Participants were randomly assigned into one of three groups: control (n = 23), sham tDCS (n = 29), or active tDCS (n = 31). The magnitude of imbalance between groups (due to various factors such as contraindication, no-shows, randomization miscounting) here would be considered small and would not have a meaningful effect on the primary analysis (i.e., unequal variances across groups). The inclusion of a control group (i.e., a group that did not receive either sham or active tDCS) is important for the detection of a possible placebo effect^38^. Importantly, participants randomized into the control group were informed of the study’s purpose (to investigate effects of tDCS on cognitive performance) during the informed consent prior to randomization, and were made aware that it would be possible they may be randomized to a group that did not receive tDCS. This is a key distinction of our study design from the double-blind, sham-controlled design often used in tDCS experiments, which helps to clarify the presence and strength of a placebo effect relative to a group that did not receive any tDCS at all. Only 5% (n = 3) of the tDCS group participants (active or sham) in this study had any previous experience with tDCS, which is consistent with the number of people who reported prior experience with tDCS among previous survey data^16^. We chose to include participants who had previous experience with tDCS since prior exposure to a treatment may also be a source of the placebo effect^14^ and would be helpful to quantify if a sufficient number of individuals with previous tDCS experience had participated. This study was registered at the ISRCTN Registry on 10/02/2023 (Registration # ISRCTN86769535, https://www.isrctn.com/ISRCTN86769535) as well as prior to recruitment on Open Science Framework (10.17605/OSF.IO/E7GW3)^39^. Informed consent was obtained from all participants prior to study participation. The study was approved by the Arizona State University Institutional Review Board (Study #00009764) and adhered to CONSORT guidelines.

### Visuospatial working memory training

All three groups completed a single 20-min session of an adapted version of the Corsi Block Tapping task (CBTT) from the Psychology Experiment Building Language (PEBL)^40^, which was used for visuospatial training. The CBTT^41^ is a visuospatial working memory task, where participants were instructed to memorize sequences of locations of squares on the screen. For any trial, nine blue squares were on the screen, and then a certain number of the squares sequentially lit up in yellow, one at a time. Participants were instructed to observe and memorize the sequence in which the blocks lit up. After the sequence was finished, participants were asked to click on the blocks in the exact sequence they had observed. The CBTT as programmed in PEBL did not have any time limit for completing each trial. Difficulty of the task differs based on the number of blocks to be memorized per trial (i.e., ‘span length’). For example, a span length of 4 indicated that 4 blocks needed to be memorized. The maximum (i.e., most difficult) possible span length was nine blocks. After completing three practice trials at a span length = 3, participants trained on the CBTT for 20 min, starting at span length = 4. The span length increased to 5 only when the participant correctly completed two consecutive trials with a span length of 4. Span length continued to increase in this manner for 20 min. If a span length of 9 was reached within the training session, all remaining trials had a span length of 9. Our primary outcome measure was span length. Although we recorded the accuracy for each trial (% of blocks correctly tapped relative to the span), this was not selected as our primary outcome measure since span lengths would only increase when two consecutive trials had 100% accuracy. Accuracy for each trial was recorded simply to verify that participants were engaged during training. Theoretically, if a participant’s accuracy dropped below 25% for a given trial, this would be considered as random guessing and may warrant trial exclusion. This did not, however, happen in any of the trials in this study.

### tDCS protocol

For this study, all participants were tested in the same room, and the physical set-up of the room remained constant across all participants, with the exception of the absence or presence of the stimulator between the control and active/sham tDCS groups, respectively. There was no difference between the active and sham tDCS groups which blinded experimenter conducted the study (χ^2^ = 7.68, df = 8, p = 0.47). All experimenters were trained by the same researcher. There was also no difference between groups in the time of day at which the data were collected (F_1,52_ = 3.84, p = 0.06). All experimenters followed the same script when administering the study, including informed consent and study description.

Each group trained for 20 min on the CBTT either with or without wearing the tDCS device. As recommended by Colloca and Barsky, the control group completed CBTT training without wearing the tDCS device on their scalp. For the active and sham tDCS groups, 20-min stimulation was administered concurrently with visuospatial training to ensure functional cortical engagement during stimulation^11^. The tDCS setup used 5 × 7 cm electrodes (Soterix Medical Inc.). The active electrode (anode) was placed on the right posterior parietal lobe (P4 on the International 10–20 System), and the return electrode (cathode) on the contralateral supraorbital area. It is important to note that placement of the cathode over the contralateral supraorbital area does not have an inhibitory effect on the frontal lobe, and has been shown to be just as effective at increasing current density of the anodal area similar to that of an extracephalic cathodal placement (i.e. contralateral shoulder or neck)^42^. The active tDCS group received 20 min of 2 mA anodal stimulation, including a 30 s ramp-up and a 30 s ramp-down at the beginning and end of the session. This montage and stimulation parameters were chosen to prioritize increasing excitability of the right posterior lobe. Prior research that has demonstrated that this montage and stimulation parameters are effective at increasing both local and global mean field power of the right posterior parietal lobe^43^. Sham stimulation was delivered via the manufacturer’s auto-sham setting, which ramps up the current intensity to 2 mA for thirty seconds and then ramped off to 0 mA at the start and end of the 20-min training session to provide similar sensory experiences between the sham and active tDCS groups for blinding purposes. These sham stimulation parameters have been effective for blinding in other studies using tDCS during visuospatial training^6,7^. A separate experimenter set up the tDCS equipment and stimulation to ensure blinding of the participants and experimenters involved in the training.

Following stimulation, participants’ expectations about the efficacy of tDCS to improve cognitive performance were measured with a question adapted from the Credibility and Expectancy Questionnaire^44^: “Do you think brain stimulation would improve your cognitive performance?”, answered with a scale of 0–8 (0 = ‘no, not at all’; 4 = ‘neutral’; 8 = ‘yes, very much.’ We asked this question at the end of the study, after training was completed, to ensure that the question itself did not prime or bias participants in advance. We also asked participants which group they believed they were assigned to (‘active’, ‘sham’, or ‘not sure’) to ensure that methods for blinding were effective. We only asked the anodal and sham tDCS groups these questions because the control group had no interaction with the tDCS device during the study, and the control group was unblinded.

To monitor potential side effects associated with stimulation, participants in the tDCS groups also answered questions from the tDCS Questionnaire before and after stimulation as recommended by Thair^45^. Participants reported their symptoms related to head, neck, and back pain, scalp sensation, feelings of fatigue, mood, and anxiety on a 10-point Likert scale (1 = no symptom presence to 10 = very severe at the moment). A total symptom score was quantified by summing across each question, with higher scores indicating more severe symptoms. A change score indicating an increase (or decrease) in negative symptoms was calculated by subtracting the post-stimulation score from the pre-stimulation score.

### Statistical analysis

We first examined the effect of active tDCS on CBTT training by using a linear mixed effects model with the span length as the dependent variable, along with the logarithm of when each CBTT trial started (log time in training, starting at 0 min), group, and a log time in training-by-group interaction as fixed effects; random intercepts by participant were modeled as random effects. The start of each span attempt within the training session was recorded in PEBL, and then transformed to its logarithm (see justification below). Due to the high variation in the number of trials that each participant performed and the pace at which they practiced, we did not perform a random slopes model due to chance of overfitting. We note that because the duration of CBTT training was constant across participants (i.e., 20 min), the number of trials and each trial duration was variable across participants. For example, if participants successfully completed the first two trials with a span length of 4, their third trial would have a span length of 5, which would take longer to complete than a participant whose third trial remained a span length of 4. Participants were also not required to complete any given trial within any specified time window. We also note that the time point of when each trial started during the 20-min session was transformed to its logarithm (referred throughout as *log time in training*) to allow for comparison across participants because participants’ span length improved more early on in training and less so later in practice; thus, a linear model would not be appropriate and a logarithmic model was a better fit (AIC_log_ = 7179, AIC_linear_ = 7350). Figure 1 illustrates this rationale. The purpose of this first analysis was simply to examine any treatment effect of active tDCS on CBTT training. This analysis was only performed on the two groups that received active or sham tDCS in the double-blind, sham-controlled tDCS study design.

Next, we performed the same analysis but with the inclusion of the control group (i.e., a group that received no tDCS device exposure). This was our a priori analysis plan, in order to first follow a standard double-blind, sham-controlled study design that compares the active and sham groups. We then collapsed the tDCS groups to yield a single group that was exposed to the tDCS device. Thus, we performed a linear mixed effects model with span length as the outcome variable, log time in training, group, and a log time in training-by-group interaction as fixed effects, and random intercepts by participant as random effects.

Our next analyses examined the extent to which (1) tDCS expectations of participants who received either active or sham tDCS and (2) their perceived stimulation influenced the change in CBTT performance during training. Since there are no well-established factors that influence tDCS-specific placebo effects, we iteratively built a linear mixed effects model with tDCS group assignment as a control variable, and participant expectation and perceived stimulation condition as our placebo-related exploratory variables. We used analysis of variance using a log-likelihood test to identify the final model that best fit the data^46^. We did so in this case (rather than using AIC values) because this allows for better estimation of statistical differences between nested models. Because the extent to which negative side-effects are experienced is also subject to placebo effects^38^, we used a linear model where the primary outcome was the change in the total symptom score from the tDCS Questionnaire from pre- to post-stimulation, such that a larger positive change indicates that a participant experienced more negative symptoms as a function of receiving tDCS (or perceiving that they received tDCS). This model included group, participants’ expectations about tDCS, and perceived stimulation condition (self-reported as “sham”, “not sure”, or “active”) as independent variables predicting change in negative symptoms.

We lastly used independent t-tests and Fisher exact tests to confirm that the active and sham tDCS groups were matched in terms of expectation and blinding (i.e., perceived stimulation condition) (Supplementary Information S1).

### Supplementary Information

Supplementary Information.

## Data Availability

Upon acceptance of this article, the datasets used for each result will be made publicly available on Zenodo, 10.5281/zenodo.7551889.
