# Supplementary material for: Evidence and sources of placebo effects in transcranial direct current stimulation during a single session of visuospatial working memory practice
Source: Sci Rep. 2024 Apr 20;14:9094. doi: 10.1038/s41598-024-59927-2 (PMC11032394; doi:10.1038/s41598-024-59927-2)
Supplement: Supplementary file 1 — Supplementary Information. [file 41598_2024_59927_MOESM1_ESM.docx]

**Research Protocol: Evidence and sources of placebo effects in transcranial direct current stimulation during visuospatial working memory training**

**Protocol ID#:** This study was registered at the ISRCTN Registry on February 10, 2023 (ISRCTN86769535) and prior to recruitment on Open Science Framework

**Name of Investigators**

Andrew Hooyman

Nicole Haikalis

Peiyuan Wang

Heidi M. Schambra

Keith R. Lohse

Sydney Y. Schaefer

**Responsible for conducting research**

Nicole Haikalis and Peiyuan Wang

**Research site address**

611 E. Orange St., Room 158D

Tempe, AZ 85281

**Research site telephone number**

480-727-6651

**Name and address of sponsor/funder**

NASPSPA Graduate Student Research Grant – address available upon request

Arizona State Global Sport Institute – address available upon request

Arizona State University Graduate College JumpStart Program – 1120 Cady Mall, Tempe, AZ 85281

**Project Summary**

The primary aim of this trial was to examine the effectiveness of transcranial direct current stimulation (tDCS) to improve performance during visuospatial working memory training among non-disabled young adults. We also emphasized analysis of potential placebo effects which may result in improved performance in visuospatial working memory that was independent from the treatment effect of tDCS but was related to the participants expectation that tDCS would improve their visuospatial working memory performance. Therefore, the overall objectives of the study were to investigate the direct treatment effect of tDCS on visuospatial memory but also placebo effects on performance related to tDCS. The trial recruited 3 groups (Active tDSC, Sham tDCS, and a no-tDCS control) to examine the presence and magnitude of tDCS treatment and placebo effects. The inclusion of non-tDCS control group allowed us to compare possible tDCS related placebo effects on performance to what would be considered typical performance improvements absent any treatment or placebo effects of tDCS. The study was conducted over a 11 month period (1/20/2021 – 12/2/2021) in which participants that met the study’s inclusion criteria were randomly assigned to one of the three trial groups. Participants who were assigned to one of the tDCS groups were blinded to which type of tDCS they received (Active or Sham). Participants in the tDCS groups were given a questionnaire pre and post visuospatial memory training that measured their expectancies of tDCS to improve their performance on the task. Participant’s negative side effects associated with receiving tDCS were also collected pre and post practice.

**Rationale and Background information**

The use of tDCS as a treatment modality to improve both clinical and behavioral outcomes in humans has been widely investigated. However, there is no clear consensus on the efficacy of tDCS in either a broad or specific context. Interestingly, a tDCS device can be constructed by individuals from the general public using common materials found at a local hardware store. It is a more known and used experimental device than perhaps of other medical devices under scientific investigation. Due to the greater potential widespread knowledge and use of tDCS it may be more likely that beneficial effects of tDCS use are more associated with placebo rather than treatment effects. Therefore, determination of the effectiveness of tDCS as a treatment is imperative to better understand its appropriate use in both medical and general populations.

**Study Goals and Objectives**

The primary goal of this study is to determine if tDCS effectively improves visuospatial performance among non-disabled young adults and if any observed improvements in performance are related to the treatment or placebo effect of tDCS. Our main objective is to quantify differences in performance between groups who receive either active, sham or no tDCS and then analyze if expectancies associated with tDCS are related to between and within group differences in performance.

**Study Design**

This study is designed as a double-blind clinical trial focused on human behavior. The population of interest for recruitment into the trial was young non-disabled adults. All participants in this study had no self-reported history of mental illness, neurologic disease, or injury (i.e., stroke, history of seizures, concussion diagnosis, brain disease, or arthritis of the hands or upper limbs). All participants reported normal visual acuity and the absence of any peripheral sensory or motor loss/pathology. Participants enrolled into the study completed one session of tDCS and visuospatial training or just visuospatial training which took 2 hours during a single day of data collection.

**Methodology**

*Inclusion Criteria*

All participants in this study had no self-reported history of mental illness, neurologic disease, or injury (i.e., stroke, history of seizures, concussion diagnosis, brain disease, or arthritis of the hands or upper limbs). All participants reported normal visual acuity and the absence of any peripheral sensory or motor loss/pathology.

*Randomization*

Participants that were enrolled in the experiment were assigned to one of three groups (Active tDCS, Sham tDCS, and no-tDCS) using simple randomization.

*Blinding*

Participants who were assigned to the tDCS groups were not told if they were receiving active or sham tDCS. A separate research staff member, other than the one directly interacting with the participant, set the tDCS device to either active or sham and did not disclose which stimulation was selected for the participant to the interacting research staff member.

*Visuospatial working memory training*

All three groups completed a single 20-minute session of an adapted version of the Corsi Block Tapping task (CBTT) from the Psychology Experiment Building Language (PEBL) [35], which was used for visuospatial training. The CBTT is a visuospatial working memory task, where participants were instructed to memorize sequences of locations of squares on the screen. For any trial, nine blue squares were on the screen, and then a certain number of the squares sequentially lit up in yellow, one at a time. Participants were instructed to observe and memorize the sequence in which the blocks lit up. After the sequence was finished, participants were asked to click on the blocks in the exact sequence they had observed. The CBTT as programmed in PEBL did not have any time limit for completing each trial. Difficulty of the task differs based on the number of blocks to be memorized per trial (i.e., ‘span length’). For example, a span length of 4 indicated that 4 blocks needed to be memorized. The maximum (i.e., most difficult) possible span length was nine blocks. After completing three practice trials at a span length = 3, participants trained on the CBTT for 20 minutes, starting at span length = 4. The span length increased to 5 only when the participant correctly completed two consecutive trials with a span length of 4. Span length continued to increase in this manner for 20 minutes. If a span length of 9 was reached within the training session, all remaining trials had a span length of 9. Our primary outcome measure was span length. Although we recorded the accuracy for each trial (% of blocks correctly tapped relative to the span), this was not selected as our primary outcome measure since span lengths would only increase when two consecutive trials had 100% accuracy. Accuracy for each trial was recorded simply to verify that participants were engaged during training. Theoretically, if a participant’s accuracy dropped below 25% for a given trial, this would be considered as random guessing and may warrant trial exclusion. This did not, however, happen in any of the trials in this study.

*tDCS protocol*

For this study, all participants were tested in the same room, and the physical set-up of the room remained constant across all participants, except for the absence or presence of the stimulator between the control and active/sham tDCS groups, respectively. All experimenters were trained by the same researcher. All experimenters followed the same script when administering the study, including informed consent and study description.

Each group trained for 20 minutes on the CBTT either with or without wearing the tDCS device. The control group completed CBTT training without wearing the tDCS device on their scalp. For the active and sham tDCS groups, 20-minute stimulation was administered concurrently with visuospatial training to ensure functional cortical engagement during stimulation. The tDCS setup used 5x7 cm electrodes (Soterix Medical Inc.). The active electrode (anode) was placed on the right posterior parietal lobe (P4 on the International 10-20 System), and the return electrode (cathode) on the contralateral supraorbital area. The active tDCS group received 20 minutes of 2 mA anodal stimulation, including a 30 sec ramp-up and a 30 sec ramp-down at the beginning and end of the session. At the beginning and end of the sham stimulation period, the current intensity was ramped on to 2mA and ramped off to 0 mA over 30 seconds. As stated previously, a separate experimenter set the tDCS equipment and stimulation up to ensure blinding of the participants and experimenters involved in the training.

Following stimulation, participants’ expectations about the efficacy of tDCS to improve cognitive performance were measured with a question adapted from the Credibility and Expectancy Questionnaire: “Do you think brain stimulation would improve your cognitive performance?”, answered with a scale of 0-8 (0 = ‘no, not at all’; 4 = ‘neutral’; 8 = ‘yes, very much.’ We asked this question at the end of the study, after training was completed, to ensure that the question itself did not prime or bias participants in advance. We also asked participants which group they believed they were assigned to (‘active’, ‘sham’, or ‘not sure’) to ensure that methods for blinding were effective. We only asked the anodal and sham tDCS groups these questions because the control group had no interaction with the tDCS device during the study, and the control group was unblinded.

To monitor potential side effects associated with stimulation, participants in the tDCS groups also answered questions from the tDCS Questionnaire before and after stimulation as recommended by Thair [38]. Participants reported their symptoms related to head, neck, and back pain, scalp sensation, feelings of fatigue, mood, and anxiety on a 10-point Likert scale (1 = no symptom presence to 10 = very severe at the moment). A total symptom score was quantified by summing across each question, with higher scores indicating more severe symptoms. A change score indicating an increase (or decrease) in negative symptoms was calculated by subtracting the post-stimulation score from the pre-stimulation score.

**Study Design Graphic**

**
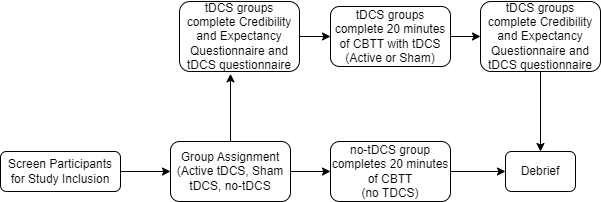
**

**Safety Considerations**

All participants were monitored by research staff for any discomfort or any other adverse event during the experiment. In the event that a participant would be put in danger as a result of tDCS the research staff member would be able to shut down the device and remove it from the participant without incident. All research staff were trained in appropriate research ethics.

**Follow-up**

No follow-up was performed after the study was completed. The application of tDCS at the levels used in this protocol have been found to be safe with any long term adverse events being extremely rare.

**Data management and statistical analysis**

Data are handled within a secure, dual factor authenticated, cloud based server. Data were analyzed using R software. We used linear mixed effects models to analyze our primary research questions: Is there evidence of tDCS treatment or placebo effects on visuospatial working memory? Do tDCS expectancies explain tDCS related changes in performance independent of tDCS treatment effects. Our primary outcome variable was span length, i.e. total number of correct blocks tapped, during each trial of the Corsi Block Tapping Task. Our primary independent variables were group (Active tDCS, Sham tDCS, and no-tDCS), time at which each trial commenced during the 20 minute practice block, and tDCS expectancies. Group was coded as a categorical variable (dummy coded), trial time was coded as a continuous variable in milliseconds, and expectancies were coded as an interval variable (Likert scale). Within our mixed effects model we analyzed a group by trial time interaction to determine if there was a main effect of group, i.e. any initial difference between groups at the start of practice, and if the rate of improvement differed between groups, i.e. if one group improved their performance at a higher rate than the other groups. In a follow-up analysis, among only the tDCS groups, we examined if tDCS expectancies were related to changes in performance. Specifically, we modeled a trial time by expectancy interaction whereby the level of individual expectancy would relate to either a higher or lower rate of performance improvement on the Corsi Block Tapping Task.

**Quality Assurance**

All paper forms, i.e. informed consent, documents were stored in a secure location within the laboratory. All digital data were stored and backed up to a dual authenticated cloud-based server. All data were deidentified to protect the identity of the participants.

**Expected outcomes**

We hypothesized that differences in visuospatial performance would exist between the Active and Sham tDCS groups and between the Sham tDCS and no-tDCS groups. Additionally, we expect that tDCS expectancies among the tDCS groups would explain some magnitude of performance change independent of tDCS specific treatment effects.

**Duration of the Project**

Data collection was completed over a 11-month period. Compilation of data and analysis of results was completed over a 3 month period. Drafting and editing of the manuscript was completed over a 4 month period.

**Problems anticipated**

The primary problem faced to the completion of this research was the continued waves of COVID-19 during the data collection period.

**Ethics**

All research staff were trained in the ethical and responsible conduct of research (CITI). All data were deidentified so as to protect the identities of the participants.
